# A nomogram based on psoas muscle index predicting long-term cirrhosis incidence in non-cirrhotic patients with HBV-related acute‑on‑chronic liver failure

**DOI:** 10.1038/s41598-023-47463-4

**Published:** 2023-12-02

**Authors:** Jie Bai, Manman Xu, Fengling Peng, Junwei Gong, Xiaodong Song, Yongguo Li

**Affiliations:** 1 Department of Infectious Diseases, The First Affiliated Hospital of Chongqing Medical University, Chongqing, China; 2 Fourth Department of Liver Disease (Difficult & Complicated Liver Diseases and Artificial Liver Center), Beijing You’an Hospital Affiliated to Capital Medical University, Beijing, China; 3 Department of Radiology, The First Affiliated Hospital of Chongqing Medical University, Chongqing, China; 4 Department of Neurology, The First Affiliated Hospital of Chongqing Medical University, Chongqing, China

**Keywords:** Medical research, Hepatology

## Abstract

There is a lack of scoring system to predict the occurrence of cirrhosis in individuals with acute-on-chronic liver failure (ACLF) in the absence of cirrhosis. The goal of this study was to develop a psoas muscle index (PMI)-based nomogram for cirrhosis risk in non-cirrhotic patients with HBV-related ACLF. We included 274 non-cirrhotic HBV-ACLF patients who were randomly assigned to training and validation groups. Logistic analyses were performed to identify risk factors for cirrhosis. A nomogram was then constructed. The predictive performance of the nomogram was assessed using the area under the receiver operating characteristic curve (AUROC), calibration curve, and decision curve analysis (DCA). During the 360-day follow-up, 44.5% (122/274) of non-cirrhotic HBV-ACLF patients developed cirrhosis. A higher PMI at the L3 level was correlated with a decreased risk of long-term cirrhosis occurrence (OR 0.677, 95% CI 0.518–0.885, P = 0.004). The nomogram incorporating PMI, age, neutrophil-to-lymphocyte ratio (NLR), and international normalized ratio (INR), indicated satisfactory predictive performance for cirrhosis risk stratification in ACLF population. The nomograms had an AUROC of 0.812 (95% CI 0.747–0.866) and 0.824 (95% CI 0.730–0.896) in the training and validation cohorts, respectively. The calibration curves displayed excellent predictive accuracy of the nomogram in both sets. In both cohorts, the DCA verified the nomogram's clinical efficacy. In non-cirrhotic HBV-ACLF patients, a greater PMI appears to protect against long-term cirrhosis occurrence. Strong predictive performance has been demonstrated by PMI-based nomograms in assessing the likelihood of 1-year cirrhosis in those with HBV-ACLF.

## Introduction

Acute-on-chronic liver failure (ACLF) is a condition characterized by acute and severe liver decompensation in patients with underlying chronic liver disease (CLD), with or without cirrhosis^1^. In Western countries, alcoholic cirrhosis is the leading cause of ACLF, while in the Asia–Pacific region, hepatitis B virus (HBV) infection is the main underlying condition^1, 2^. The prognosis of ACLF is generally poor, with up to a 40% 28-day mortality rate^3^. While some non-cirrhotic ACLF patients may recover completely, others may gradually progress to cirrhosis, leading to impaired quality of life and increased mortality. Therefore, identifying risk factors for cirrhosis development in this population is crucial for developing preventive or therapeutic strategies. Current scoring systems, such as the model for end-stage liver disease (MELD) and MELD-Na scores, are useful for measuring disease severity and determining organ allocation^1, 4, 5^. However, they have not been validated for predicting cirrhosis development in ACLF patients. Additionally, no scoring system has yet incorporated indicators that reflect the nutritional status of ACLF patients without cirrhosis.

Sarcopenia, which refers to the loss of skeletal muscle mass and function, is a major component of the malnutrition in patients suffering liver disease^6^. A growing body of research has linked sarcopenia to poor prognosis in various CLD^7–12^. Recent evidence has also highlighted the importance of sarcopenia in predicting short-term outcomes and long-term mortality in ACLF population^10, 13^. However, there is currently no research investigating the potential role of sarcopenia in predicting long-term incidence of cirrhosis in ACLF patients without cirrhosis.

At present, computed tomography (CT) measurements of the muscle at the third lumbar vertebra (L3) have been utilized to diagnose sarcopenia, as skeletal muscle mass at this level is thought to be representative of whole-body skeletal muscle mass^14–16^. Thus, we aimed to investigate the potential association between the L3 psoas muscle index (PMI) and the long-term development of cirrhosis in HBV-ACLF patients without cirrhosis, and subsequently established a novel nomogram based on the PMI.

## Methods

### Patients

Patients with HBV-ACLF who were admitted at the First Affiliated Hospital of Chongqing Medical University between January 2012 and August 2021 were included in this study retrospectively. The following were the inclusion requirements: (1) 18 years and over; (2) hepatitis B surface antigen (HBsAg) and/or HBV DNA positive for more than 6 months; (3) ACLF is diagnosed using the Asia–Pacific Association for the Study of the Liver (APASL) criteria^1^, which includes jaundice (total bilirubin [TB]) ≥ 85.5 μmol/L) and coagulation disorder (international normalized ratio [INR] ≥ 1.5) as well as ascites and/or hepatic encephalopathy (HE) within 4 weeks; (4) received an abdominal CT while in the hospital; and (5) had been followed up for at least 360 days.

Patients who fit any of the following descriptions were ineligible: (1) ACLF combined with cirrhosis; (2) diagnosis of hepatocellular carcinoma (HCC) or other cancers; (3) concurrent consumptive diseases such as tuberculosis or hyperthyroidism; (4) undergoing prolonged corticosteroid treatment; (5) malnutrition due to neuromuscular disease or prolonged bedridden state; (6) death or receiving a liver transplant; and (7) significant weight changes in the short term.

The Ethics Committee of the First Affiliated Hospital of Chongqing Medical University approved this observational retrospective cohort study (2022-K434) with a waiver of patients' informed consent. The study was carried out in accordance with the Declaration of Helsinki.

### Clinical data

Demographic data (gender, age, height, and weight), laboratory findings (routine blood, liver function, renal function, electrolytes, coagulation indices, and HBV virologic markers), and antiviral nucleoside analogue usage for HBV throughout follow-up were all clinical data that we gathered. We calculated the prognostic scores for MELD and MELD-Na using pertinent data collected at the time of admission.

A thorough evaluation of the clinical, biochemical, radiographic (including ultrasound, elastography, CT, and MRI) and pertinent endoscopic findings linked to cirrhosis and/or portal hypertension, as well as the results of a liver biopsy, is required for the diagnosis of cirrhosis^17^. The doctors used these standards to assess the cirrhosis data for all recruited patients at baseline and 1 year after enrollment.

### Evaluation of Psoas Muscle Index

Within 2 weeks of the diagnosis of ACLF, all enrolled patients underwent abdominal CT scans manufactured by Siemens AG, Germany. Two imaging specialists independently evaluated the lumbar muscle area (cm^2^) at the L3 cross-section of the CT image using the 3D Slicer program (version 5.1.0). A third doctor was brought in to resolve any differences that arose. The patient's height (m^2^) divided by the area of the psoas muscle at the L3 level was used to compute PMI^14, 15^.

### Statistical analysis

MedCalc software (version 20.1.0), IBM SPSS Statistics (version 22.0), and R software (version 4.1.2) were used for statistical analyses. The study comprised 274 patients, with 183 randomly allocated to the training cohort to generate the prediction model and the remaining 91 patients assigned to the validation group to test model performance. Quantitative data that were normally distributed were subjected to the unpaired t-test, whereas non-normally distributed data were subjected to Mann–Whitney U tests. The comparisons between the categories of categorical variables were analyzed using the Chi-square test.

For the training cohort, univariate logistic regression analysis was used to find putative cirrhosis predictors. The multivariate regression model only contained variables that had a P value 0.05 in the univariate analysis. An independent predictor-based nomogram was created in the R software, utilizing the rms library. Utilizing the area under the receiver operating characteristic curve (AUROC), the discriminatory performance of the developed models was compared. Model calibration was performed by plotting calibration curves of true probabilities against model predicted probabilities. To determine net benefits, decision curve analysis (DCA) was performed. The threshold for statistical significance was set at P < 0.05.

## Results

### Patients' baseline characteristics

A total of 274 HBV-ACLF patients without cirrhosis were included in this study. Patients' mean PMI was 6.4 ± 1.5 cm^2^/m^2^, and their average age was 46.4 ± 13.8 years overall (Table S1). The mean MELD and MELD-Na scores were 24.5 ± 4.1 and 24.9 ± 4.1, respectively. ACLF susceptibility factors for all patients were detailed in Table S2. During the follow-up period of at least 1 year, cirrhosis was diagnosed in 122 (44.5%) patients.

Two groups of 274 patients, one for training (n = 183) and the other for validation (n = 91), were randomly allocated. Regarding baseline characteristics, the two groups were similar (Table S1). The incidence of cirrhosis was found to be comparable in the training and validation cohorts [44.3% (81/183) vs. 45.1% (41/91), P = 0.901)].

### Factors involved in progression to cirrhosis

In the training cohort, 81 (44.3%) patients developed cirrhosis within 360 days, and 102 (55.7%) patients were not affected with cirrhosis. Table 1 presents the demographics data, laboratory information, complications, MELD, and MELD-Na scores of the two groups. Patients who progressed to cirrhosis were older than those who did not (51.2 ± 13.9 vs. 42.2 ± 12.9 years, P < 0.001). In the cirrhosis group, the proportion of hepatic encephalopathy (HE) was significantly higher (24.7% vs. 2.0%, P < 0.001). Besides, compared with the non-cirrhotic population, patients in the cirrhosis group displayed higher white blood cell count (WBC), neutrophil-to-lymphocyte ratio (NLR), international normalized ratio (INR), and total bilirubin (TB), but lower levels of albumin (ALB), cholinesterase (ChE), serum sodium (Na), and alpha-fetoprotein (AFP) (P < 0.05). Compared to the non-cirrhotic group, individuals with cirrhosis exhibited substantially lower L3 PMI (5.8 ± 1.5 cm^2^/m^2^ vs. 6.8 ± 1.5 cm^2^/m^2^, P < 0.001). Furthermore, patients with ACLF who progressed to cirrhosis had significantly higher MELD scores (26.4 ± 5.0 vs. 23.4 ± 3.0, P < 0.001) and MELD-Na scores (27.0 ± 4.9 vs. 23.6 ± 3.0, P < 0.001) than those who did not. There was no statistically significant difference in Child–Pugh scores between the two groups (P = 0.240).

**Table 1:** Comparison of the baseline characteristics between cirrhosis and non-cirrhosis groups in the training cohort.

Variable	Non-cirrhosis group (n = 102)	Cirrhosis group (n = 81)	P-value
Male, n (%)	85 (83.3)	67 (82.7)	0.912
Age (years), mean (SD)	42.2 (12.9)	51.2 (13.9)	< 0.001
BMI (kg/m^2^), mean (SD)	23.2 (3.1)	22.9 (3.5)	0.574
PMI (cm^2^/m^2^), mean (SD)	6.8 (1.5)	5.8 (1.5)	< 0.001
Virological data			
HBsAg (IU/mL), median (IQR)	4399.0 (652.0–25,000.0)	1991.0 (162.6–19,689.1)	0.152
HBeAg positive, n (%)	47 (46.1)	35 (43.2)	0.698
HBV DNA (log_10_ IU/mL)			0.618
< 4.0	22 (21.6)	20 (24.7)	
≥ 4.0	80 (78.4)	61 (75.3)	
Use of antiviral therapy, n (%)	94 (92.2)	74 (91.4)	0.845
WBC (×10^9^/L), median (IQR)	5.8 (4.8–7.3)	6.7 (4.9–8.6)	0.037
NLR, median (IQR)	3.2 (2.3–4.4)	4.7 (3.4–6.7)	< 0.001
HB (×10^9^/L), mean (SD)	134.1 (18.9)	130.4 (18.5)	0.187
PLT (×10^9^/L), median (IQR)	134.0 (93.8–164.8)	130.0 (91.5–158.5)	0.559
INR, median (IQR)	1.8 (1.7–2.1)	2.1 (1.8–2.5)	< 0.001
TB (μmol/L), median (IQR)	222.2 (157.1–301.2)	267.0 (183.3–373.7)	0.007
ALB (g/L), mean (SD)	33.5 (6.3)	31.0 (5.0)	0.003
ALT (U/L), median (IQR)	803.0 (359.3–1602.8)	675.0 (219.0–1268.5)	0.154
AST (U/L), median (IQR)	525.5 (210.3–853.3)	554.0 (237.5–1000.0)	0.414
GGT (U/L), median (IQR)	150.0 (95.8–190.3)	108.0 (83.0–176.5)	0.088
ALP (U/L), median (IQR)	148.0 (126.8–178.4)	160.0 (128.0–196.3)	0.153
ChE (U/L), median (IQR)	3839.5 (3153.7–4621.6)	3378.0 (2759.9–4244.9)	0.029
Na (mmol/L), mean (SD)	139.2 (2.9)	137.6 (4.6)	0.007
CR (μmol/L), median (IQR)	66.0 (58.0–73.3)	66.0 (55.5–79.0)	0.990
Child–Pugh score, mean (SD)	10.1 (1.8)	10.4 (1.8)	0.240
MELD score, mean (SD)	23.4 (3.0)	26.4 (5.0)	< 0.001
MELD-Na score, mean (SD)	23.6 (3.0)	27.0 (4.9)	< 0.001
AFP (ng/mL), median (IQR)	139.2 (42.0–255.5)	59.2 (18.4–106.3)	< 0.001
Hepatic encephalopathy, n (%)	2 (2.0)	20 (24.7)	< 0.001
Ascites, n (%)	61 (59.8)	61 (75.3)	0.027

SD, standard deviation; IQR, interquartile range; BMI, body mass index; PMI, psoas muscle index; WBC, white blood cell count; NLR, neutrophil-to-lymphocyte ratio; HB, hemoglobin; PLT, platelet; INR, International normalized ratio; TB, total bilirubin; ALB, albumin; ALT, alanine aminotransferase; AST, alanine aminotransferase; GGT, glutamyl transpeptidase; ALP, alkaline phosphatase; ChE, cholinesterase; Na, Serum sodium; CR, serum creatinine; MELD, model for end-stage liver disease; AFP, alpha-fetoprotein.

We performed univariate and multivariate logistic regression analysis to find possible variables connected to the long-term development to cirrhosis in HBV-ACLF patients. Age, PMI, WBC, NLR, INR, TB, ALB, ChE, Na, and AFP were significantly linked with progression to cirrhosis in ACLF patients, according to the results of the univariate analysis (P < 0.05), as presented in Table 2. Table 2 also includes the findings of the multivariate logistic analysis, which demonstrated that patients with HBV-ACLF had a lower risk of developing cirrhosis if they had higher PMI levels after 360 days of follow-up, with an odds ratio (OR) of 0.677 (95%CI 0.518–0.885, P = 0.004). In addition, age, NLR, and INR all contributed to an increased incidence of cirrhosis (P < 0.05).

**Table 2:** Univariate and multivariate regression analysis of cirrhosis in the training cohort.

Variable	Univariate		Multivariate	
	OR (95% CI)	P-value	OR (95% CI)	P-value
Age (years)	1.051 (1.027–1.076)	< 0.001	1.039 (1.011–1.068)	0.006
PMI (cm^2^/m^2^)	0.633 (0.505–0.793)	< 0.001	0.677 (0.518–0.885)	0.004
WBC (×10^9^/L)	1.134 (1.017–1.264)	0.023		
NLR	1.390 (1.194–1.617)	< 0.001	1.465 (1.220–1.760)	< 0.001
INR	2.621 (1.507–4.556)	0.001	2.748 (1.474–5.124)	0.001
TB (μmol/L)	1.004 (1.001–1.006)	0.003		
ALB (g/L)	0.912 (0.857–0.971)	0.004		
ChE (U/L)	1.000 (0.999–1.000)	0.040		
Na (mmol/L)	0.891 (0.821–0.968)	0.006		
AFP (ng/mL)	0.999 (0.997–1.000)	0.043		

BMI, body mass index; PMI, psoas muscle index; WBC, white blood cell count; NLR, neutrophil-to-lymphocyte ratio; HB, hemoglobin; PLT, platelet; INR, International normalized ratio; TB, total bilirubin; ALB, albumin; ALT, alanine aminotransferase; AST, alanine aminotransferase; GGT, glutamyl transpeptidase; ALP, alkaline phosphatase; ChE, cholinesterase; Na, Serum sodium; CR, serum creatinine; AFP, alpha-fetoprotein; OR, odds ratio; CI, confidence interval.

### Construction of PMI-based nomogram for long-term progression to cirrhosis

**Figure 1:**
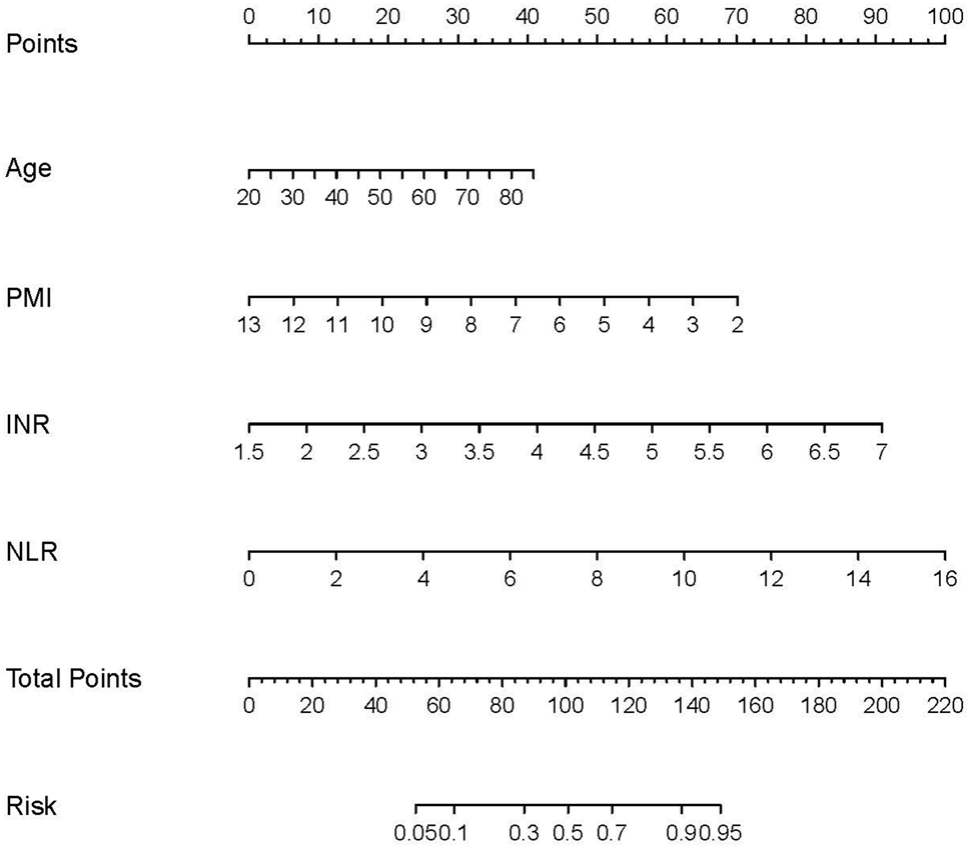
The nomogram used to predict the risk of cirrhosis in the training cohort. PMI, psoas muscle index; NLR, neutrophil-to-lymphocyte ratio; INR, International normalized ratio.

We then developed a nomogram using the four independent predictors mentioned above, namely PMI, age, NLR, and INR (Fig. 1). The patient's score was determined by summing the scores from the four risk variables, which were obtained using the vertical axis of each risk indicator. The probability of developing cirrhosis for each individual was then acquired from the "Total Points" axis of the nomogram.

### Validation of PMI-based nomogram

**Figure 2:**
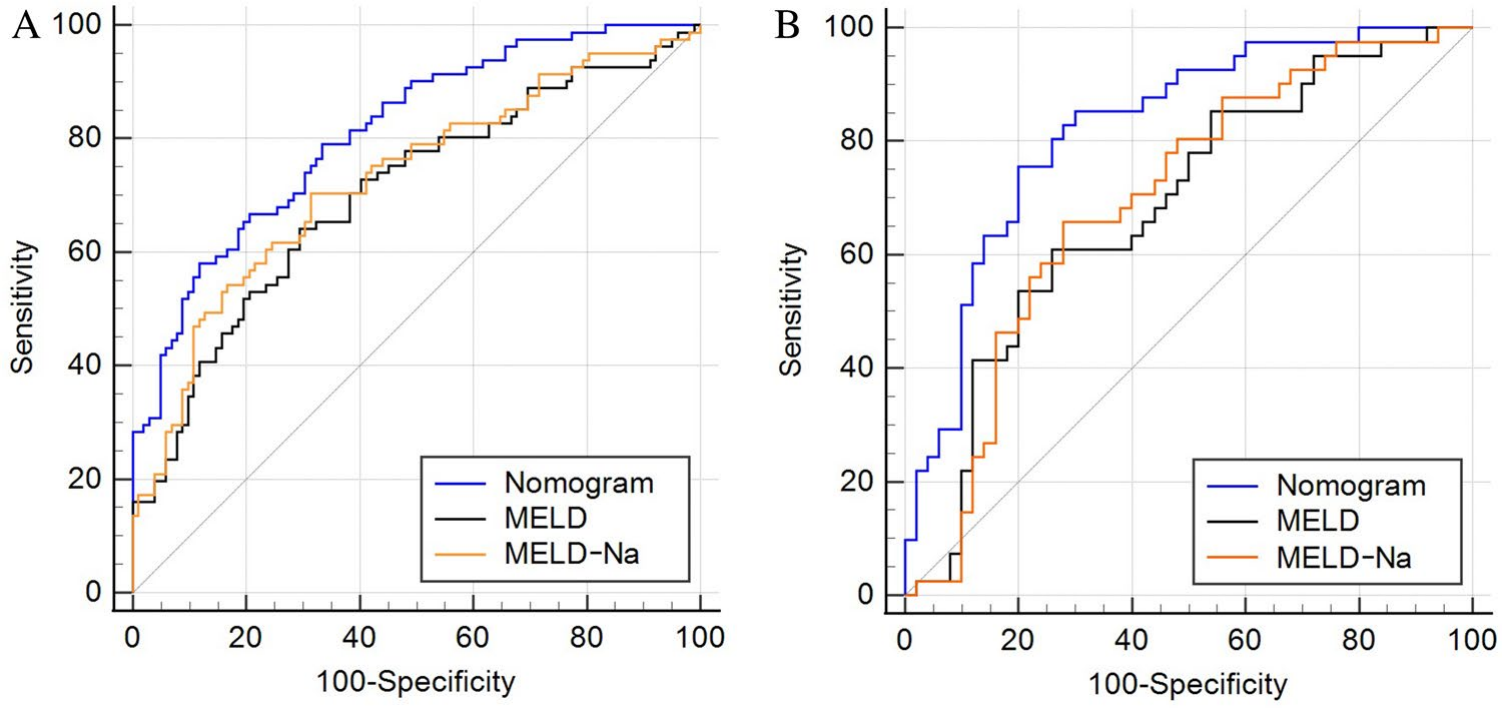
Area under the receiver operating characteristic curve (AUROC) for nomogram, MELD and MELD-Na scores in the training set (**A**) and validation set (**B**). MELD, Model for end-stage liver disease.

**Figure 3:**
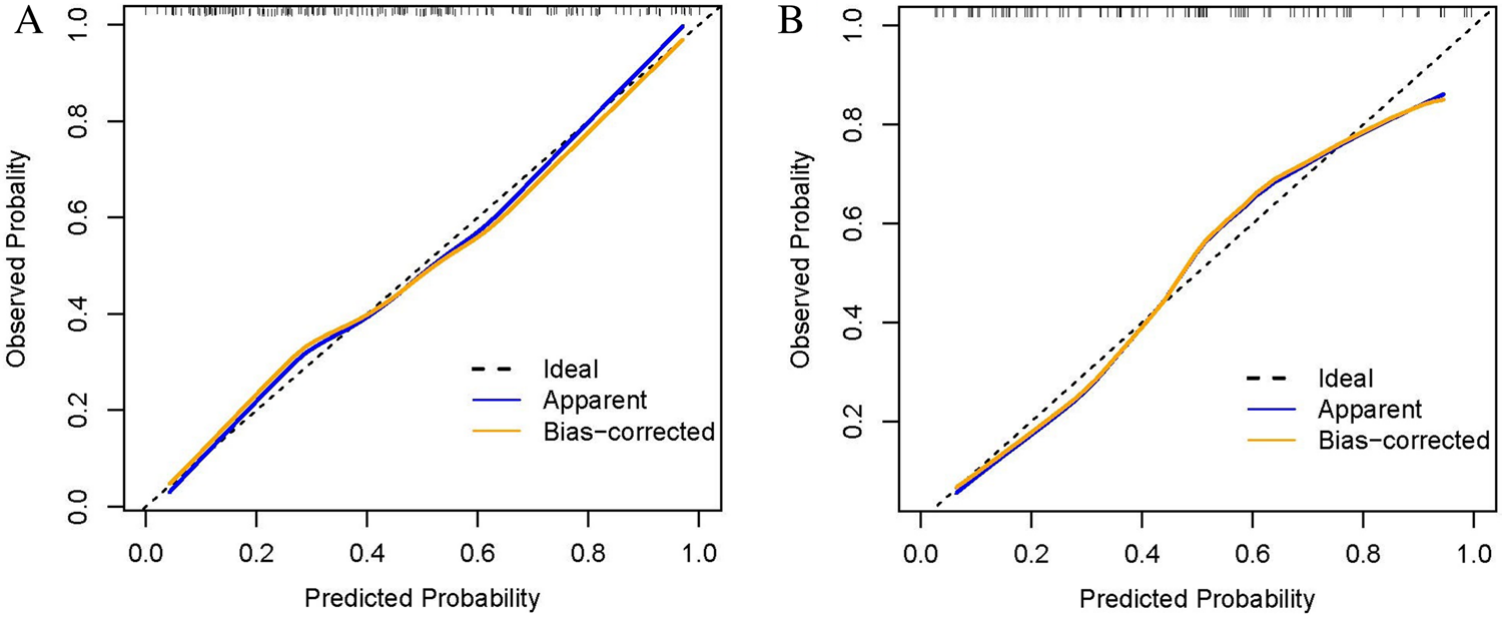
The calibration curve of nomogram. (**A**) Mean absolute error was found to be 0.021 for the training set; (**B**) Mean absolute error was noted to be 0.026 for the validation cohort.

**Figure 4:**
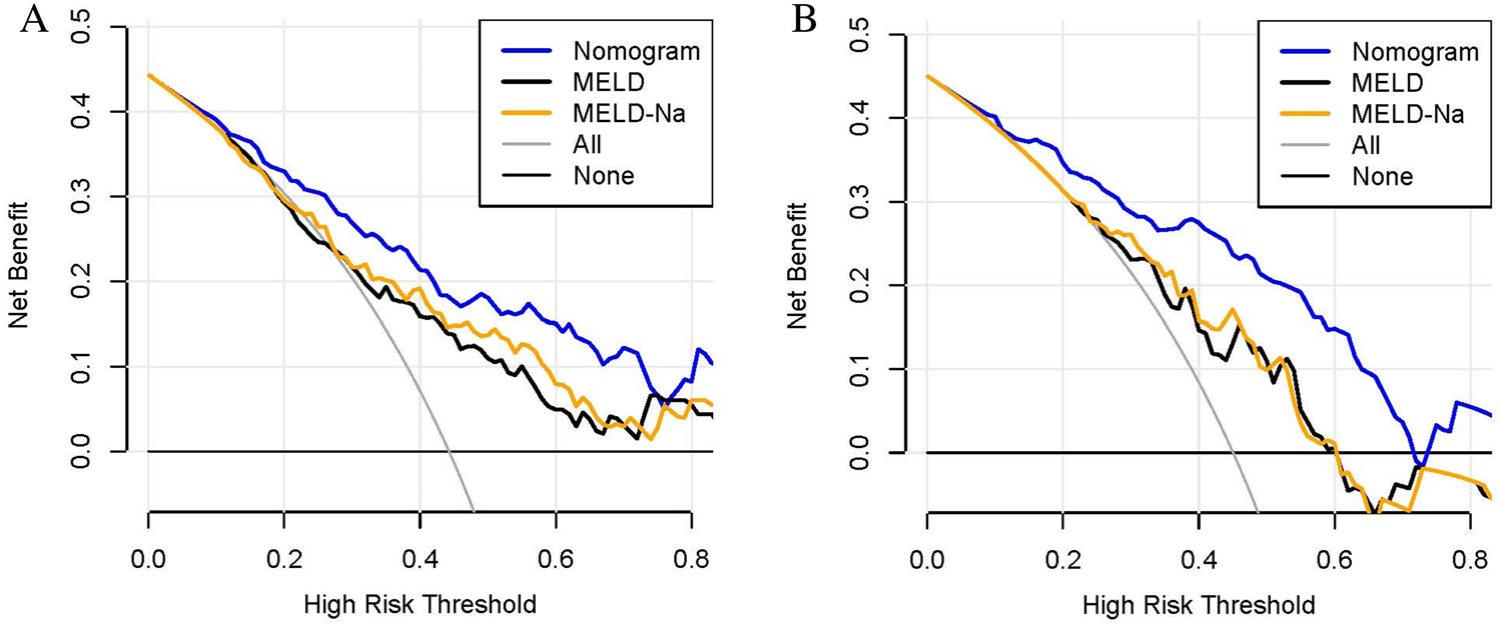
The decision curves of the nomogram, MELD and MELD-Na scores in the training set (**A**) and validation set (**B**). MELD, Model for end-stage liver disease.

The nomogram outperformed the MELD and MELD-Na scores in the training group, having significantly higher AUROCs [0.812 (95% CI 0.747–0.866), 0.702 (95% CI 0.630–0.768), and 0.727 (95% CI 0.656–0.790), respectively, both P < 0.05], demonstrating superior discriminatory capacity (Fig. 2A). Similar outcomes were obtained from the validation set, with the AUROCs for the nomogram, MELD, and MELD-Na scores being 0.824 (95% CI 0.730–0.896), 0.686 (95% CI 0.580–0.779), and 0.697 (95% CI 0.592–0.789), respectively, all with P values < 0.05 (Fig. 2B). The calibration curves for the validation and training sets both displayed good model effectiveness, with close agreement between predicted and observed probabilities of cirrhosis, as evidenced by a mean absolute error of 0.021 and 0.026, respectively (Fig. 3A,B). With threshold probabilities of > 3% and > 4%, respectively, the decision curve analysis (DCA) revealed a satisfactory net benefit of using the nomogram for predicting cirrhosis in both the training and validation cohorts (Fig. 4A,B).

## Discussion

To our knowledge, this is the first research to exam how PMI could affect non-cirrhotic HBV-ACLF patients' long-term (1 year) progression to cirrhosis. Our research suggests that a higher PMI at L3 may prevent the development of cirrhosis. We have also developed a PMI-based nomogram for cirrhosis risk stratification in HBV-ACLF patients. This nomogram demonstrated good predictive performance for the occurrence of cirrhosis in non-cirrhotic HBV-ACLF patients at baseline. By weighing the likelihood of cirrhosis occurrence, these patients can be identified in advance and individualized clinical management could be optimized during the recovery.

Despite the fact that there is no consensus definition for ACLF, it is widely acknowledged to be a complicated clinical condition marked by abrupt hepatic deterioration in CLD, along with organ failure and substantial short-term death rates^18–20^. The Asia Pacific consortium has defined ACLF as acute liver failure following acute hepatic insult in patients with underlying CLD or cirrhosis^1^. However, while many prior studies have concentrated primarily on mortality in ACLF individuals, liver reserve during recovery and long-term clinical outcomes in survivors, has been overlooked largely. In fact, due to severe acute liver decompensation and imbalance in injury as well as regeneration, a subset of non-cirrhotic ACLF patients can gradually develop irreversible cirrhosis with quality of life and longevity impacted. There are currently no scoring models for non-cirrhotic ACLF patients that can predict the long-term incidence of cirrhosis. In this study, we explored various risk variables affecting long-term (1-year) progression to cirrhosis in HBV-ACLF patients. Our findings suggest that the risk of long-term cirrhosis in HBV-ACLF individuals is substantially linked to increased age, NLR, and INR as well as lower PMI.

Sarcopenia, a form of malnutrition, can affect up to 70% of advanced liver disease patients^21^. In recent years, sarcopenia's impact on patients with CLD has gained significant attention. Studies have established a strong correlation between sarcopenia and liver disease severity^9, 22–24^, with sarcopenia effectively predicting unfavorable outcomes, particularly in cirrhosis patients^7, 25, 26^. Sarcopenia has been associated to liver fibrosis in those with non-alcoholic fatty liver disease (NAFLD)^27, 28^. Patients with significant fibrosis have lower SMI than those without^29^; low SMI acted as an independent predictor of advanced liver fibrosis^29, 30^. Recent studies have also shown that lower PMI or SMI at L3 level is highly correlated with increased mortality risk in ACLF patients^10, 11, 13^. In non-cirrhotic HBV-ACLF patients, our research revealed a link between PMI and long-term cirrhosis development. The results remained consistent after taking into account additional significant variables in a multifactorial logistic regression analysis.

The underlying biological mechanism linking sarcopenia to ACLF progression remains incompletely understood. However, evidence suggests that ACLF is accompanied by intense systemic inflammation and oxidative stress, which can disrupt the delicate equilibrium between protein synthesis and breakdown^31, 32^. Additionally, hyperammonemia resulting from severe liver dysfunction can lead to muscle wasting through the upregulation of myostatin, an important myokine involved in the muscle-liver crosstalk^6^. This crosstalk involves the secretion of various cytokines and proteins by skeletal muscle that may affect other tissues, including the liver^33^. Studies have shown that myostatin activates hepatic stellate cells via the JNK signaling pathway, which leads to liver fibrosis^34^. Other molecular factors, such as irisin and vitamin D, also significantly impact muscle-liver crosstalk^35^. Therefore, muscle dysfunction could accelerate the advancement of liver disease, including ACLF, and low PMI may be a contributing factor to poor outcomes, such as progression to cirrhosis.

Age is a primary and immutable risk factor for the advancement of CLD, such as NAFLD, hepatic fibrosis, cirrhosis, and liver cancer^36, 37^. Liver ageing can exacerbate necrotic apoptosis and chronic inflammation in the liver, which in turn leads to liver fibrosis and other CLD^38^. NLR is a simple and well-defined marker of immune dysregulation. High NLR level indicates poor prognosis of ACLF after the liver transplantation^39^. It has been reported that among cirrhotic individuals, a higher NLR is linked to a 9% rise in the probability of death within a year^40^. It is possible that the predictive power of NLR addresses multiple pathways in the pathogenesis of CLD, involving the triggering of mild endotoxemia, and eliciting detrimental systemic inflammatory response. Thus, aging and high NLR levels can lead to progression to cirrhosis in ACLF patients not affected with cirrhosis. INR serves as a key index for diagnosing ACLF, reflecting both the liver injury and coagulation abnormality. Our findings are consistent with a few previous studies that found a possible connection between INR and adverse outcomes due to liver failure^1, 41, 42^. Additionally, INR was shown to be able to predict fibrosis in individuals with chronic hepatitis B^43^. Our findings provide additional evidence that INR contribute to cirrhosis development in ACLF subjects without pre-existing cirrhosis.

In this study, we discovered that PMI-based nomograms outperformed MELD and MELD-Na scores at predicting the long-term risk of cirrhosis in patients with non-cirrhotic HBV-ACLF. This may be explained by the fact that MELD and MELD-Na scores are designed for those with severe end-stage liver disease, while our study included non-cirrhotic ACLF patients. This could also be due to the strong association reported between sarcopenia and cirrhosis, which was neglected by MELD and MELD-Na scores. The nomogram can effectively guide the frequency of the follow-up and the decision related to initiation of nutritional intervention. Higher nomogram scores might lead to recommendations for more frequent follow-up and more aggressive nutritional intervention for greater benefit. Our DCA results indicated that more patients with HBV-ACLF could benefit from the timely and appropriate treatment when the threshold probability of the nomogram was > 3%.

The current study has certain limitations. First, it was retrospective in nature, thus resulting in inherent bias in the results. Second, all the subjects and their clinical information were obtained from a single center and external validation was lacking. Third, this study only analyzed the possible effect of baseline PMI on outcome, but the dynamic alterations of PMI were not taken into consideration. Lastly, the liver status prior to ACLF, particularly the extent of liver fibrosis, affects the risk of cirrhosis during the recovery period. Unfortunately, this study lacks data on this aspect. Therefore, the impact of PMI on the prognosis of ACLF lacks adequate assessment. Hence, multicenter prospective studies could be performed in future to further verify the robustness and applicability of our conclusions.

In summary, our study revealed that among non-cirrhotic HBV-ACLF patients, a lower PMI is independently correlated with the development of cirrhosis. The PMI-based nomogram can be a useful tool for assessing the likelihood of cirrhosis in non-cirrhotic HBV-ACLF patients at 1 year.

### Supplementary Information

Supplementary Tables.

## Data Availability

The datasets generated during and/or analysed during the current study are available from the corresponding author on reasonable request.
