# Supplementary material for: A nomogram based on psoas muscle index predicting long-term cirrhosis incidence in non-cirrhotic patients with HBV-related acute‑on‑chronic liver failure
Source: Sci Rep. 2023 Dec 2;13:21265. doi: 10.1038/s41598-023-47463-4 (PMC10692120; doi:10.1038/s41598-023-47463-4)
Supplement: Supplementary file 1 — Supplementary Tables. [file 41598_2023_47463_MOESM1_ESM.docx]

**Table S1 The baseline characteristics of all patients**

| **Variable** | **All cases**  **(n = 274)** | **Training set**  **(n = 183)** | **Validation set**  **(n = 91)** | **P-value** |
| --- | --- | --- | --- | --- |
| Male, n (%) | 224 (81.8) | 152 (83.1) | 72 (79.1) | 0.427 |
| Age (years), mean (SD) | 46.4 (13.8) | 46.2 (14.1) | 46.9 (13.3) | 0.708 |
| BMI (kg/m²), mean (SD) | 23.0 (3.6) | 23.1 (3.3) | 22.9 (4.1) | 0.788 |
| PMI (cm^2^/m^2^), mean (SD) | 6.4 (1.5) | 6.4 (1.6) | 6.4 (1.4) | 0.938 |
| Virological data |  |  |  |  |
| HBsAg (IU/mL), median (IQR) | 4240.3 (640.2-23517.2) | 3846.2 (463.3-22923.5) | 4960.4 (1184.5-25000.0) | 0.095 |
| HBeAg positive | 129 (47.1) | 82 (44.8) | 47 (51.6) | 0.285 |
| HBV DNA (log_10_ IU /mL) |  |  |  | 0.698 |
| <4.0 | 61 (22.3) | 42 (23.0) | 19 (20.9) |  |
| ≥4.0 | 213 (77.7) | 141 (77.0) | 72 (79.1) |  |
| Use of antiviral therapy | 255 (93.1) | 168 (91.8) | 87 (95.6) | 0.243 |
| WBC (×10^9^/L), median (IQR) | 6.2 (4.9-7.9) | 6.1 (4.8-7.9) | 6.6 (4.9-8.1) | 0.344 |
| NLR, median (IQR) | 3.7 (2.5-5.5) | 3.7 (2.6-5.6) | 3.7 (2.5-5.2) | 0.687 |
| HB (×10^9^/L), mean (SD) | 132.6 (18.8) | 132.4 (18.8) | 133.0 (19.0) | 0.833 |
| PLT (×10^9^/L), median (IQR) | 132.0 (94.8-167.0) | 134.0 (93.0-161.0) | 130.0 (102.0-171.0) | 0.857 |
| INR, median (IQR) | 1.9 (1.7-2.3) | 1.9 (1.7-2.3) | 1.9 (1.7-2.4) | 0.575 |
| TB (μmol/L), median (IQR) | 237.2 (163.4-328.7) | 245.0 (170.0-336.2) | 222.7 (153.5-321.5) | 0.365 |
| ALB (g/L), mean (SD) | 32.5 (5.6) | 32.4 (5.9) | 32.8 (5.0) | 0.622 |
| ALT (U/L), median (IQR) | 705.5 (279.5-1356.5) | 756.0 (312.0-1483.0) | 518.0 (241.0-1280.0) | 0.247 |
| AST (U/L), median (IQR) | 525.5 (204.0-937.5) | 539.0 (218.0-947.0) | 408.0 (179.0-936.0) | 0.282 |
| GGT (U/L), median (IQR) | 133.5 (91.8-190.3) | 133.0 (91.0-184.0) | 134.0 (92.0-210.0) | 0.402 |
| ALP (U/L), median (IQR) | 153.3 (125.0-189.0) | 153.0 (127.0-187.0) | 157.0 (123.6-196.0) | 0.967 |
| ChE (U/L), median (IQR) | 3707.5 (2956.8-4587.1) | 3705.0 (2956.0-4428.0) | 3762.0 (2957.0-4721.0) | 0.410 |
| Na (mmol/L), mean (SD) | 138.5 (3.7) | 138.5 (3.8) | 138.5 (3.6) | 0.922 |
| CR (μmol/L), median (IQR) | 65.0 (56.0-74.0) | 66.0 (57.0-74.0) | 64.0 (51.0-72.0) | 0.139 |
| Child-Pugh score, mean (SD) | 10.2 (1.8) | 10.3 (1.8) | 10.0 (1.8) | 0.267 |
| MELD score, mean (SD) | 24.5 (4.1) | 24.7 (4.3) | 24.2 (3.8) | 0.314 |
| MELD-Na score, mean (SD) | 24.9 (4.1) | 25.1 (4.3) | 24.5 (3.9) | 0.293 |
| AFP (ng/mL), median (IQR) | 84.0 (27.2-187.4) | 83.0 (23.8-187.3) | 85.0 (36.3-199.1) | 0.672 |
| Hepatic encephalopathy, n (%) | 35 (12.8) | 22 (12.0) | 13 (14.3) | 0.597 |
| Ascites, n (%) | 179 (65.3) | 122 (66.7) | 57 (62.6) | 0.509 |

SD, standard deviation; IQR, interquartile range; BMI, body mass index; PMI, psoas muscle index; WBC, white blood cell count; NLR, neutrophil-to-lymphocyte ratio; HB, hemoglobin; PLT, platelet; INR, international normalized ratio; TB, total bilirubin; ALB, albumin; ALT, [alanine aminotransferase](https://www.abbreviationfinder.org/cn/acronyms/alt_alanine-amino-transferase.html); AST, alanine aminotransferase; GGT, glutamyl transpeptidase; ALP, [alkaline phosphatase](https://www.abbreviationfinder.org/cn/acronyms/balp_bone-alkaline-phosphatase.html); ChE, cholinesterase; Na, Serum sodium; CR, serum creatinine; MELD, model for end-stage liver disease; AFP, alpha-fetoprotein.

**Table S2. The predisposing factors for all patients with HBV-ACLF**

| **Variable** | **All cases**  **(n = 274)** | **Training set**  **(n = 183)** | **Validation set**  **(n = 91)** | **P-value** |
| --- | --- | --- | --- | --- |
| Predisposing factors, n (%) |  |  |  | 0.913 |
| Reactivation of HBV | 190 (69.3) | 127 (69.4) | 63 (69.2) |  |
| Alcohol | 12 (4.4) | 7 (3.8) | 5 (5.5) |  |
| Bacterial infection | 42 (15.3) | 28 (15.3) | 14 (15.4) |  |
| Drugs or Poisons | 25 (9.1) | 18 (9.8) | 7 (7.7) |  |
| Unclear | 5 (1.8) | 3 (1.6) | 2 (2.2) |  |
